# Public priorities for local action to reduce the health impacts of climate change: Evidence from a UK survey

**DOI:** 10.1016/j.puhip.2022.100346

**Published:** 2022-11-20

**Authors:** Alexander Harrison, Hilary Graham

**Affiliations:** University of York, United Kingdom

**Keywords:** 2015 Paris agreement, Public perspectives, Local government, Air pollution, Floods

## Abstract

**Objectives:**

To investigate public concerns about the impacts of climate change on people's health in the UK and their priorities for action by local government. In the UK, local government are responsible for the environmental protection and health of their local population.

**Study design:**

Cross-sectional survey.

**Methods:**

An online survey of UK adults aged ≥18 years was conducted in 2021 (n = 4050). Representative quotas were set for gender, age group, ethnic group, educational attainment and location (UK country/England region). Survey participants were asked about their concerns about the health impacts of climate change and, excluding those reporting no concerns, their top priorities for their local government to address.

**Results:**

The dominant health concerns related to air pollution and severe floods. These exposures were also identified as the two most important priorities for local government to address. Separate logistic regression models investigated local-level factors that predicted the selection of each priority, taking account of socio-demographic factors. For both outcomes, awareness of the relevant exposure in the local area in the past 12 months doubled the odds of selecting it as a priority (air pollution: OR 2.01, 95%CI 1.71, 2.36; floods: OR 2.16, 95%CI 1.88, 2.48).

**Conclusions:**

The study demonstrates the potential of surveys to capture public priorities for local action on the health impacts of climate change, and to yield clear policy advice on the issues of greatest public concern.

## Highlights

• Increasing emphasis is being given to local-level climate policies informed by the views of the public.
• Evidence on people's concerns and priorities for local action is scant.
• In a UK survey, the major concerns about the health impacts of climate change related to air pollution and severe floods.
• These two exposures were also identified as the major priorities for local government to address.
• The study points to the potential for local strategies to be informed by public concerns and priorities.

## 1 Introduction

Protecting people's health from climate change requires policies that address climate-related exposures like flooding, air pollution and heatwaves [1,2]. The 2015 Paris Agreement instituted a ‘bottom-up’ climate governance regime, through which national governments develop their own plans for emissions reduction and climate adaptation [3]. This decentralised structure enables climate policy to take account of public concerns and priorities [4]. A global review found that public support for climate action increased when linked to local issues like air pollution [4] and, in turn, local support is critical for policy adoption at local level [5]. However, little is known about people's priorities for action by local government.

We investigate this question through a UK study. Local government in the UK is responsible for environmental protection and the health of their local community, and the national government is promoting local-level interventions to address climate change [6]. Our study is based on surveys conducted in 2021 before (October) and after (December) the 26th UNFCCC Conference of Parties (COP26) held in the UK, a period of heightened media engagement in climate change.

## 2 Methods

Adults aged ≥18 years were recruited via an online UK panel of people aged ≥18 years who have agreed to be contacted about participation in surveys.^1^ Representative quotas were set for gender, age group, ethnic group, educational attainment and location (UK country/England region). Ethical approval was secured from the Department of Health Sciences, University of York (ref: HSRGC/2020/409/C).

The response options for the question on concerns about the health impacts of climate change (Table 1) were developed in an earlier qualitative study. Excluding those reporting no concerns, a follow-up question asked ‘Thinking about these problems and the harmful impacts they may have on people's health, what are your top priorities for your local government to address?’ Using the same list (Table 1), participants were asked to select their first and second priority. In a separate section of the survey, participants were asked about their awareness of ‘any of the following in your local area in the past 12 months’, with the response options of ‘flooding, heatwave (where people's health is significantly affected), coastal erosion, air pollution (poor air quality), none of the above’. They were additionally asked if they had ‘personally experienced’ any of these exposures in the past 12 months.

**Table 1:** Question on the health impacts of climate change.

Thinking about the harmful impacts that climate change may have on people's health in the UK, what kind of changes in the climate concern you? You can select more than one.
Response options^a^
• Air pollution (poor air quality)
• Severe storms
• Drought (a prolonged period without rain)
• Severe floods
• Heat waves
• Coastal erosion (where the sea wears away the land)
• Increasing temperatures
• Sea level rises
• Wildfires
• Other – please describe
• None of these concern me

a The order of the first nine response options was randomised.

Our analysis focuses on the two priorities most frequently selected. In separate models that took account of sociodemographic factors (gender, age group, ethnic group, educational attainment, tenure, health status), we investigated local-level factors with the potential to influence these priorities: type of area (urban/outskirts of town or city/small village/rural) and awareness of the relevant exposure ‘in your local area’ in the previous 12 months. Personal experience of the exposure in the previous 12 months, climate change concern and survey month were entered as covariates. Backward stepwise logistic regression (inclusion set to p < 0.1) included all factors, with non-significant variables sequentially removed until the final was model was reached.

## 3 Results

Participants were recruited prior to (October, n = 2040) and after (December, n = 2010) COP26. Table S1 describes the sample profile.

In response to the question about concerns about the health impacts of climate change (Table 1), the most frequently reported were air pollution (67%) and severe floods (68%) (Table S2). Over 50% of participants also noted concerns about severe storms (61%), sea level rise (56%), increasing temperatures (54%), coastal erosion (54%) and heatwaves (53%). A small minority (5%, n = 219) reported no concerns. The remaining sample (n = 3831) were asked for their priorities for local government action.

**Fig. 1:**
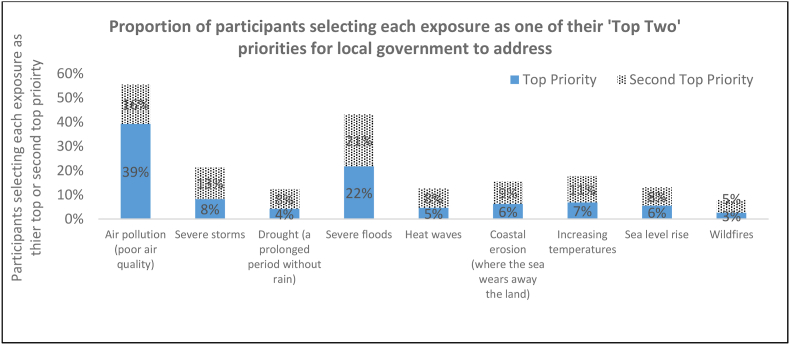
Top two priorities for their local government to protect people in the UK from the health impacts of climate change (n = 3831).

Air pollution and severe floods were identified as top priorities by 55% and 43% of participants respectively (Fig. 1), with no significant differences by survey month (Table S3). There was some overlap in these groups: of those selecting air pollution as their top priority, 31% (n = 462) selected floods as their second priority. Of those placing floods top, 33% (n = 279) selected air pollution as their second priority.

In both regression models (Tables S4 and S5), local-level factors were significant predictors of selecting air pollution and severe floods, respectively, as top priorities for local-government action. In a model (Table S4) that took account of individual experience of air pollution, awareness of local-area air pollution in the previous 12 months doubled the odds of selecting it as a top priority (OR 2.01, 95%CI 1.71, 2.36). Similarly, awareness of local-area flooding doubled the odds of severe floods being a top priority (Table S5; OR 2.16, 95%CI 1.88, 2.48). Type of residential area was also significant for both outcomes. Compared to those in urban areas, participants in rural areas had a reduced likelihood of prioritising air pollution. For flooding, participants living in the outskirts of a town/city or in small villages prioritised severe floods in greater likelihood to those in urban areas.

Sociodemographic predictors varied between the selected priorities. Being female and living in rented/other housing tenure increased the odds of selecting air pollution as a top priority for local action (Table S4). For severe floods (Table S5), being older and having high educational attainment increased the odds of selecting this priority for action; being from a black and minority ethnic group reduced the likelihood. In neither model was survey month significant, pointing to a stability in people's priorities across a period of heightened media engagement in climate change.

The models were a good fit with all factors significant in the final step also being significant at the first step, providing reassurance the associations and findings are robust.

## 4 Discussion

The 2015 Paris Agreement instituted a decentralised system of global climate governance, with nations determining their own climate action plans [3]. As part of this shift, local communities and local tiers of government are playing a greater role in action on climate change [4]. However, little is known about public concerns and priorities for local action.

Our study focuses on the UK where, with central government funding of local authorities falling by 75% since 2010 [7], the concerns and priorities of local communities are assuming greater importance. The study design also enabled account to be taken of any ‘COP26 effect’ on public concern about the health impacts of climate change and their priorities for local action.

Some study limitations should be noted. The surveys were conducted before the UK's heatwaves in June–August 2022; a later survey may have captured heightened public concern about this exposure. The study's areal measures were limited to country/English region and type of area. It lacked an area-level deprivation measure, but included domains from which these measures are derived (education, employment, housing, health) [8]. Participants' awareness of local air pollution and flooding (Table S1) could not be geo-referenced; however, national monitoring data for PM₂.₅ levels and flooding confirm a high national prevalence of risk [9,10]. While most (95%) UK adults have access to the internet, participant recruitment via an online survey platform excluded those without access, a group facing multiple disadvantages.

Our study points to widespread public concern about the health impacts of climate change, particularly air pollution and floods, exposures which are adversely affecting health in the UK [1,10]. The study underlines the importance of local experiences in shaping people's priorities for action by local government. Their prioritisation of air pollution and floods relates directly to the twin pillars of climate policy to protect public health [2]. With air pollution primarily driven by fossil fuel consumption, reducing climate-damaging emissions (mitigation) is the key policy lever, while reducing vulnerability to climate effects (adaptation) is central to protecting people against flooding.

In conclusion, our study points to a broad public consensus on the priorities for local action in the UK: air pollution and severe floods. The study also demonstrates the potential of surveys to deliver clear policy messages on issues of public concern.

## Funding

This research was funded by NIHR Public Health-Policy Research Programme, grant number PR_PRU_1217_20901. The study is independent research carried out by the NIHR Public Health Policy Research Unit (PH-PRU), commissioned and funded by the National Institute for Health Research Policy Research Programme. The views expressed in the report are those of the authors and not necessarily those of the NHS, the National Institute for Health and Care Research, the Department of Health and Social Care or its arm’s length bodies, and other Government Departments.

## Declaration of competing interest

The authors declare no conflicts of interest. The funders had no role in the design of the survey, in the collection, analyses, or interpretation of data; in the writing of the manuscript, or in the decision to publish the results.

## Footnotes

1 Qualtrics. *Research services*. 2021. Available from: https://www.qualtrics.com/uk/research-services/.
