# Supplementary material for: Public priorities for local action to reduce the health impacts of climate change: Evidence from a UK survey
Source: Public Health Pract (Oxf). 2022 Nov 20;4:100346. doi: 10.1016/j.puhip.2022.100346 (PMC9694065; doi:10.1016/j.puhip.2022.100346)
Supplement: Multimedia component 1 [file mmc1.docx]

**SUPPLEMENTARY TABLES**

**Table S1: Sample profile (n=4050)**

| **Variable** | **Recoded Category (if applicable)** | **UK Population and Quota** | **Survey Population (n=4050)** |
| --- | --- | --- | --- |
|  |  | **%** | **Column N %** |
| **Survey** | **First survey (08.10.21-20.10.21)** | N/A | 50.37 |
|  | **Second survey (09.12.21-22.12.21)** |  | 49.63 |
| **Gender** ***** | **Male** | 48.77 | 45.65 |
|  | **Female** | 51.23 | 54.35 |
| **Age Group** | **<35** | 31.33 | 26.67 |
|  | **35-54** | 38.12 | 40.02 |
|  | **55+** | 30.55 | 33.31 |
| **Ethnic Group**** | **White** | 86.00 | 87.14 |
|  | **Black & minority ethnic groups** | 14.00 | 12.86 |
| **Education question recoded into ISCED** | **Level 1 - None to GCSE D-G** | 21.06 | 16.94 |
|  | **Level 2 - GCSE A-C to Higher Education Qualification** | 40.17 | 43.63 |
|  | **Level 3 - Degree Level** | 38.77 | 39.43 |
| **Combined Region** | **Northern England (North West, North East, Yorkshire & the Humber)** | 23.33 | 34.65 |
|  | **Mid England (West Midlands, East Midlands & East of England)** | 25.26 | 24.49 |
|  | **Greater London and Southern England (South West & South East)** | 15.67 | 24.41 |
|  | **Scotland, Northern Ireland or Wales** | 8.43 | 16.45 |
| **Housing Tenure** | **Owner occupier** | N/A | 56.69 |
|  | **Rent or other** |  | 43.31 |
| **How concerned, if at all, are you about climate change?** | **Not at all to not very concerned** | N/A | 14.74 |
|  | **Fairly concerned** |  | 44.72 |
|  | **Very concerned** |  | 40.54 |
| **Local Exposure to flooding***** | **No** | N/A | 66.22 |
|  | **Yes** |  | 33.78 |
| **Local Exposure to air pollution***** | **No** |  | 69.43 |
|  | **Yes** |  | 30.57 |
| **Personal Exposure to flooding****** | **No** |  | 92.40 |
|  | **Yes** |  | 7.60 |
| **Personal Exposure to air pollution****** | **No** |  | 79.28 |
|  | **Yes** |  | 20.72 |

* as well as male and female, the question included the option of ‘prefer to self-define’ and ‘prefer not to share this information’. A small proportion (n=33; 0.8%) selected one of these options. Because of small numbers, gender was included as a binary (male/female) category in the analysis and this group was not included

** response options: White—includes any White background; Mixed or multiple ethnic groups—includes White and Black Caribbean, White and Black African, White and Asian, or any other Mixed ethnic group; Asian or Asian British—includes Indian, Pakistani, Bangladeshi, Chinese, or any other Asian background; Black, African, Caribbean, or Black British—includes African, Caribbean, or any other Black background; Other—for example Arab or any other

*** asked at the end of the survey: ‘Are you aware of any of the following in your local area in the past 12 months?’ Responses included flooding and air pollution (poor air quality)

**** asked at the end of the survey: ‘Have you personally experienced any of the following in the past 12 months?’ Responses included flooding and air pollution (poor air quality)

| **Table S2 'Thinking now about the harmful impacts that climate change may have on people’s health in the UK, what kind of impacts - if any - concern you? You can select more than one answer’¹ (n=4050)** | | |  |
| --- | --- | --- | --- |
|  | **Count** | **% of sample selecting choice** | |
| **Air pollution (poor air quality)** | 2732 | 67.46 | |
| **Severe storms** | 2487 | 61.41 | |
| **Drought (a prolonged period without rain)** | 1768 | 43.65 | |
| **Severe floods** | 2750 | 67.90 | |
| **Heat waves** | 2157 | 53.26 | |
| **Coastal erosion (where the sea wears away the land)** | 2171 | 53.60 | |
| **Increasing temperatures** | 2197 | 54.25 | |
| **Sea level rise** | 2285 | 56.42 | |
| **Wildfires** | 1540 | 38.02 | |
| **Other - please describe** | 50 | 1.23 | |
|  |  |  | |
| **None of these concern me** | 219 | 5.41 | |

**¹** except for last response option

**Table S3: Priorities for local government *‘Thinking about these problems (listed in Table S2) and the harmful impacts they may have on people’s health, what are your top two priorities for your local government to address?’* (n=3831)¹**

| **Priority for Local Government (top or second priority)** | | **Survey** | | | | | | **p-value** |
| --- | --- | --- | --- | --- | --- | --- | --- | --- |
|  |  | **First Launch (08.10.21-20.10.21)** | | **Second Launch (09.12.21-22.12.21)** | | **Total** | |  |
|  |  | **Count** | **Column N %** | **Count** | **Column N %** | **Count** | **Column N %** |  |
| **Air Pollution** | **No** | 842 | 49.30 | 866 | 50.70 | 842 | 49.30 | 0.121 |
|  | **Yes** | 1094 | 51.53 | 1029 | 48.47 | 1094 | 51.53 |  |
| **Severe Storms** | **No** | 1609 | 53.42 | 1403 | 46.58 | 1609 | 53.42 | 0.001 |
|  | **Yes** | 327 | 39.93 | 492 | 60.07 | 327 | 39.93 |  |
| **Drought** | **No** | 1679 | 50.00 | 1679 | 50.00 | 1679 | 50.00 | 0.079 |
|  | **Yes** | 257 | 54.33 | 216 | 45.67 | 257 | 54.33 |  |
| **Severe Floods** | **No** | 1094 | 50.37 | 1078 | 49.63 | 1094 | 50.37 | 0.849 |
|  | **Yes** | 842 | 50.75 | 817 | 49.25 | 842 | 50.75 |  |
| **Heatwaves** | **No** | 1670 | 49.88 | 1678 | 50.12 | 1670 | 49.88 | 0.03 |
|  | **Yes** | 266 | 55.07 | 217 | 44.93 | 266 | 55.07 |  |
| **Coastal Erosion** | **No** | 1638 | 50.56 | 1602 | 49.44 | 1638 | 50.56 | 949 |
|  | **Yes** | 298 | 50.42 | 293 | 49.58 | 298 | 50.42 |  |
| **Increasing Temperatures** | **No** | 1572 | 49.94 | 1576 | 50.06 | 1572 | 49.94 | 0.126 |
|  | **Yes** | 364 | 53.29 | 319 | 46.71 | 364 | 53.29 |  |
| **Sea Level Rise** | **No** | 1684 | 50.54 | 1648 | 49.46 | 1684 | 50.54 | 0.908 |
|  | **Yes** | 252 | 50.50 | 247 | 49.50 | 252 | 50.50 |  |
| **Wildfires** | **No** | 1778 | 50.33 | 1755 | 49.67 | 1778 | 50.33 | 0.362 |
|  | **Yes** | 158 | 53.02 | 140 | 46.98 | 158 | 53.02 |  |
| **Other Climate Effects** | **No** | 1922 | 50.62 | 1875 | 49.38 | 1922 | 50.62 | 0.206 |
|  | **Yes** | 14 | 41.18 | 20 | 58.82 | 14 | 41.18 |  |

**¹** Excludes 219 participants who reported no concerns about the health impacts of climate change

***Table S4: Logistic regression model of factors associated with identifying air pollution as a top priority¹ for local government to take action on. (n=3831)***

|  | **Odds Ratio** | **Sig.** | **95% C.I.*** | |  |
| --- | --- | --- | --- | --- | --- |
|  |  |  | **Lower** | **Upper** |  |
| **Gender (reference group: male)** |  |  |  |  |  |
| **Female** | 1.454 | 0.000 | 1.273 | 1.661 |  |
| **Housing tenure (reference group: own home)** |  |  |  |  |  |
| **Renting or other** | 1.223 | 0.003 | 1.069 | 1.400 |  |
| **Residential Area (reference group: urban)** |  |  |  |  |  |
| **Outskirts of town or city** | 1.138 | 0.096 | 0.977 | 1.325 |  |
| **Small village** | 0.998 | 0.988 | 0.805 | 1.239 |  |
| **Rural** | 0.695 | 0.004 | 0.541 | 0.892 |  |
| **Reported air pollution in local area in last 12 months (reference group: not reported)** |  |  |  |  |  |
| **Local air pollution in last 12 months** | 2.011 | 0.000 | 1.711 | 2.364 |  |
| **Reported personal experience of air pollution in last 12 months (reference group: not reported)** |  |  |  |  |  |
| **Personal exposure to air pollution in last 12 months** | 1.417 | 0.000 | 1.178 | 1.705 |  |
| **Constant** | 0.680 | 0.000 |  |  |  |
| *CI = Confidence interval | | | | | |
| Correctly predicted 60.8%, Hosmer Lemeshow 0.068, Nagelkerke r squared 0.075 | | | | | |

¹ first or second priority

***Table S5 Logistic regression model of factors associated with identifying severe floods as a top priority***¹ ***for local government to take action on. (n=3831)***

|  | **Odds ratio** | **Sig.** | **95% C.I.*** | | |
| --- | --- | --- | --- | --- | --- |
|  |  |  | **Lower** | **Upper** | |
| **Age group (reference group: <35 years)** |  |  |  |  | |
| **35-54 years** | 1.393 | 0.000 | 1.177 | 1.648 | |
| **55+ years** | 1.668 | 0.000 | 1.391 | 2.001 | |
| **Ethnic group (reference group: white)** |  |  |  |  | |
| **Black and minority ethnic groups** | 0.695 | 0.001 | 0.561 | 0.862 | |
| **Education (reference group: Level 1)** |  |  |  |  | |
| **Level 2** | 1.030 | 0.763 | 0.851 | 1.247 | |
| **Level 3** | 1.283 | 0.013 | 1.054 | 1.561 | |
| **Climate change concern (reference group: not at all concerned or not at all/not very concerned)** |  |  |  |  | |
| **Fairly concerned** | 0.928 | 0.506 | 0.743 | 1.158 | |
| **Very concerned** | 0.797 | 0.049 | 0.636 | 0.999 | |
| **Residential Area (reference group: urban)** |  |  |  |  | |
| **Outskirts of town or city** | 1.226 | 0.011 | 1.049 | 1.434 | |
| **Small village** | 1.363 | 0.006 | 1.095 | 1.696 | |
| **Rural** | 1.124 | 0.367 | 0.872 | 1.449 | |
| **Reported severe floods in local area in last 12 months (reference group: not reported)** |  |  |  |  | |
| **Local flooding in last 12 months** | 2.157 | 0.000 | 1.879 | 2.477 | |
| **Constant** | 0.394 | 0.000 |  |  | |
| *CI = Confidence interval | | | | | |
| Correctly predicted 61.0%, Hosmer Lemeshow 0.359, Nagelkerke r squared 0.073 | | | | |  |

¹ first or second priority
